# Clinical Profile of Adult Patients Presenting With Renal Dysfunction to a Tertiary Hospital Emergency Department

**DOI:** 10.7759/cureus.21873

**Published:** 2022-02-03

**Authors:** John Masina, Muhammed Moolla, Feroza Motara, Ismail S Kalla, Abdullah E Laher

**Affiliations:** 1 Emergency Medicine, University of the Witwatersrand, Johannesburg, Johannesburg, ZAF; 2 Internal Medicine, University of the Witwatersrand, Johannesburg, Johannesburg, ZAF

**Keywords:** diabetes mellitus, hypertension, sepsis, hiv, emergency department, chronic kidney disease, acute kidney injury, renal dysfunction

## Abstract

Background

Renal dysfunction is a potentially life-threatening condition that is commonly encountered in the emergency department (ED). This study aimed to describe the clinical profile of patients presenting with renal dysfunction to a tertiary-level hospital ED.

Methods

Medical records of patients presenting to the ED with renal dysfunction over a six-month period (July-December 2017) were reviewed. A descriptive analysis of the data was performed.

Results

Serum creatinine levels were measured in 7,442 (69.9%) of the 10,642 patients that were triaged into the ED. Of these, 208 (2.8%) were identified with renal dysfunction, of which 192 consented to study participation. The median age of study subjects was 49.5 (IQR 38.8-63.0) years; 108 (56.3%) were male; proteinuria on urine dipsticks was demonstrated in 108 (56.3%); 72 (37.5%) were HIV-positive; 66 (39.6%) required dialysis; 11 (5.7%) were admitted to the ICU; and 59 (30.7%) died prior to hospital discharge. More patients presented with acute kidney injury (AKI) (46.9%) compared to chronic kidney disease (CKD) (27.6%) and acute on chronic kidney disease (AoCKD) (25.5%). Sepsis was the most common precipitant of AKI (42.2%) and AoCKD (30.6%), while chronic hypertension (35.8%) and diabetes mellitus (34.0%) were the most common comorbidities in subjects with CKD.

Conclusion

Patients presenting to the ED with various risk factors and comorbidities, including HIV, sepsis, hypertension, and diabetes mellitus, may have underlying renal dysfunction. ED clinicians should therefore adopt a low threshold to screen for renal dysfunction in these patients.

## Introduction

Abnormal renal function is frequently encountered in the emergency department (ED) and may be associated with significant morbidity and mortality [1]. Presentation with abnormal renal function can be categorized as acute kidney injury (AKI), chronic kidney disease (CKD), and acute on chronic kidney disease (AoCKD) [2]. Estimates of the global prevalence range from <1% to 66% for AKI [3] and 11% to 13% for CKD [4]. Specific data on the epidemiology of renal dysfunction presentations to the ED are scant. 

AKI can be defined as an abrupt deterioration in kidney function that is potentially reversible. The Kidney Disease: Improving Global Outcomes (KDIGO) AKI working group has defined three stages of AKI severity based either on the degree of acute rise in serum creatinine or on a time-based deterioration in the urine output [5]. There are notable differences and regional discrepancies between developed and developing countries with regard to the incidence and risk factors for AKI. In developed countries, AKI is predominately hospital-acquired, affecting older, critically ill patients, whereas in developing countries, sepsis, malaria, diarrheal diseases with dehydration, and herbal medications are more common precipitants of AKI [6,7].

Comparatively, CKD is described as an abnormality of kidney structure or function that persists for more than three months. The KDIGO CKD working group has classified CKD based on the underlying etiology, degree of deterioration in glomerular filtration rate (GFR), and severity of albuminuria [8]. Risk factors for CKD include diabetes mellitus, hypertension, non-steroidal inflammatory drugs (NSAIDs), other nephrotoxic drugs, advanced age, HIV, and cardiac failure [9]. Since both low glomerular filtration rate as well as proteinuria have been associated with AKI, AoCKD is a common feature in patients with underlying CKD [10], with the adjusted odds ratio for developing AKI being significantly higher in patients with a lower GFR [11]. 

With the high prevalence of HIV infection, diabetes mellitus, cardiac diseases, hypertension, and sepsis in South Africa, the prevalence of renal dysfunction is relatively high [12,13]. Since most published studies have been conducted in the nephrology ward and the intensive care unit (ICU) settings, there is a paucity of data pertaining to the profile of presentation of renal dysfunction in the ED setting. Hence, the aim of this study was to determine the profile of clinical presentation pertaining to demographic characteristics, presenting features, renal ultrasonographic findings, requirement for renal replacement therapy, disposition, and in-hospital mortality of patients presenting to our ED with renal dysfunction.

## Materials and methods

This was a retrospective review of the medical records of 192 consecutive patients who were prospectively identified. Patients older than 18 years and presenting with renal dysfunction to the Charlotte Maxeke Johannesburg Academic Hospital Emergency Department (CMJAH ED) from July 01, 2017 to December 31, 2017 were enrolled in the study. CMJAH is a 1088-bed tertiary care public academic hospital that is affiliated with the University of the Witwatersrand. The hospital services a major portion of the Johannesburg inner city region and surrounding suburban areas. Permission to conduct the study was granted by the hospital manager, while ethics clearance was obtained from the Human Research Ethics Committee (Medical) of the University of the Witwatersrand (clearance certificate no. M170716).

For the purpose of this study, renal dysfunction was defined as the presence of either AKI, CKD, or AoCKD. Normal serum creatinine and GFR for age were calculated as per the modification of diet in renal disease (MDRD) formula. The categorization of subjects into the AKI and CKD groups was based on the KDIGO clinical practice guidelines definitions [5,8]. The following subjects were placed in the AoCKD group: (a) subjects with underlying CKD who displayed a >10% fluctuation in their serum creatinine level during their hospital stay and had not received renal replacement therapy, and (b) subjects who had received renal replacement therapy and were deemed to have presented with AoCKD by the attending nephrologist.

Prior to the initiation of data collection, the primary investigator undertook training on the methods and principles of data abstraction from medical records. The primary investigator daily reviewed the laboratory results of all patients attending the ED over the study period. Subjects with abnormal serum creatinine levels or GFR were identified and approached for study participation. Relevant data were abstracted from the medical records of consenting subjects and entered into a specifically designed data collection sheet. Patients not consenting to study participation were excluded from the study. Patient records were followed-up until data collection was completed. The process of data collection was periodically monitored by the study supervisors. Data collected included demographic details, clinical presentation, risk factors, comorbidities, laboratory findings, ultrasonographic findings, patient disposition information, and mortality.

Patient confidentiality was strictly adhered to at all times. For each subject, patient identifying information was blocked out and replaced with a unique code. To assess inter-rater reliability, an independent individual experienced in the methods of data abstraction but blinded to the study aim re-abstracted data from a sample of 20 randomly selected medical records and compared these to the data obtained by the primary investigator. The overall Cohen's kappa coefficient (κ) was 0.83, indicating that the degree of inter-rater reliability was acceptable.

Data from the individual data collection sheets were thereafter entered into Microsoft® Excel® (Microsoft 365, Version 16.0.13029.20232, Microsoft Corporation, Redmond, WA) and analyzed. Since data were predominantly categorical in nature, findings were presented using frequency and percentage. The median and interquartile range were determined for overall subject age. Reporting of study findings was in conformance with STROBE (Strengthening the Reporting of Observational Studies in Epidemiology) guidelines [14].

## Results

Of the 18,349 patients who presented to the ED over the six-month study period, 10,642 (58.0%) were triaged into the ED. The presence of renal dysfunction was noted in 208 (2.8%) of the 7,442 (69.9%) patients in whom serum creatinine and/or GFR were determined. Sixteen (7.7%) patients did not consent to study participation; hence, a total of 192 subjects were included in the final study sample.

AKI, CKD, and AoCKD accounted for 90 (46.9%), 53 (27.6%), and 49 (25.5%) presentations, respectively. A total of 108 (56.3%) subjects were male. The median age of study subjects was 49.5 (IQR 38.8-63.0) years, with 60 (31.3%) subjects who were <40 years old. Fatigue was the most common presenting complaint in 89 (46.4%) of the subjects overall. Of the 139 (72.4%) subjects in whom urine dipstick analysis was performed, proteinuria was demonstrated in 108 (56.3%), blood in 96 (50.0%), leukocytes in 38 (19.8%), and nitrites in 26 (13.5%) subjects, respectively. HIV status was only documented in 115 (59.9%) subjects, of whom 72 (62.6%) were positive. Seventy-six (39.6%) subjects required dialysis during their hospital stay; 11 (5.7%) were admitted to the ICU; and 59 (30.7%) died prior to hospital discharge. None of the study subjects died in the ED. Details of all of the above are described in Table 1.

**Table 1:** Characteristics of study subjects AKI: acute kidney injury, CKD: chronic kidney disease, AoCKD: acute on chronic kidney disease

	AKI (n=90)	CKD (n=53)	AOCKD (n=49)	Entire cohort (n=192)
Gender				
Male	58 (64.4)	25 (47.2)	25 (51.0)	108 (56.3)
Female	32 (35.6)	28 (52.8)	24 (49.0)	84 (43.7)
Age				
<40 years	25 (27.8)	16 (30.2)	19 (38.8)	60 (31.3)
40–54 years	25 (27.8)	14 (26.4)	15 (30.6)	54 (28.1)
55–69 years	22 (24.4)	20 (37.8)	5 (10.2)	47 (24.5)
≥70 years	18 (20.0)	3 (5.6)	10 (20.4)	31 (16.1)
Presenting clinical features				
Nausea	36 (40.0)	14 (26.4)	13 (26.5)	63 (32.8)
Edema	16 (17.8)	24 (45.3)	28 (57.1)	68 (35.4)
Dyspnea	44 (48.9)	11 (20.8)	12 (24.5)	67 (33.3)
Fatigue	33 (36.7)	29 (54.7)	27 (55.1)	89 (46.4)
Confusion	24 (26.7)	4 (7.5)	12 (24.5	40 (20.8)
Urine dipstick findings				
Proteinuria	39 (43.3)	36 (67.9)	33 (67.3)	108 (56.3)
Hematuria	40 (44.4)	28 (52.8)	28 (57.1)	96 (50.0)
Leukocytes	17 (18.9)	11 (20.8)	10 (20.4)	38 (19.8)
Nitrites	10 (11.1)	8 (15.1)	8 (16.3)	26 (13.5)
No abnormalities	14 (15.6)	2 (3.8)	4 (8.2)	20 (10.4)
Urinalysis not done	28 (31.1)	14 (26.4)	11 (22.4)	53 (27.6)
HIV status				
Positive	29 (32.2)	22 (41.5)	21 (42.9)	72 (37.5)
Negative	15 (16.7)	19 (35.8)	9 (18.4)	43 (22.4)
Unknown	46 (51.1)	12 (22.6)	19 (38.8)	77 (40.1)
Disposition				
Admitted to the intensive care unit	5 (5.5)	3 (6.7)	3 (6.1)	11 (5.7)
Admitted to ward	79 (87.8)	49 (92.5)	45 (81.6)	173 (90.1)
Discharged home	6 (6.7)	1 (1.9)	1 (2.0)	8 (4.2)
Renal replacement therapy				
Received dialysis	44 (48.9)	9 (17.0)	23 (46.9)	76 (39.6)
No dialysis received	46 (51.1)	44 (83.0)	26 (53.1)	116 (60.4)
Outcome				
Died	32 (35.6)	12 (22.6)	15 (30.6)	59 (30.7)
Survived to hospital discharge	58 (64.4)	41 (77.4)	34 (69.4)	133 (69.3)

Table 2 describes the etiology/risk factors for renal dysfunction among the cohort of study patients. Sepsis was the most common precipitating factor for renal dysfunction in both the AKI (n=38, 42.2%) and AoCKD (n=18, 36.7%) groups, with urosepsis being the most common source of sepsis in both groups (AKI - n=18, 47.4%; AoCKD - n=9, 50.0%). Hypertension (n=12, 22.6%), diabetes mellitus (n=11, 20.8%), and HIV-associated nephropathy (n=8, 15.1%) were the most common precipitants of renal dysfunction in the CKD group.

**Table 2:** Etiology/risk factors for renal dysfunction amongst the cohort of study subjects ^1^Carcinoma of the cervix (n=8, 8.9%), urolithiasis (n=7, 7.8%), prostate carcinoma (n=5, 5.6%), urethral stricture (n=3, 3.3%), ovarian cancer (n=2, 2.2%) ^2^A single case each (1.1%) of acute pancreatitis, upper gastrointestinal bleed, perforated peptic ulcer, intestinal intussusception, malaria, and recent surgery ^3^A single case each (1.9%) of renal cell carcinoma, Alport syndrome, systemic lupus erythematosus and rapidly progressing glomerular nephritis ^4^Prostate carcinoma (n=4, 8.2%), benign prostate enlargement (n=3, 6.1%), carcinoma of the bladder (n=1, 2.0%)

Pathology	
AKI	n=90
Sepsis	38 (42.2)
Urosepsis	18 (20.0)
Abdominal sepsis	3 (3.3)
Pneumonia	9 (10.0)
Skin and soft tissue infections	7 (7.8)
Infective endocarditis	1 (1.1)
^1^Obstructive nephropathy	25 (27.8)
Gastroenteritis	12 (13.3)
Herbal medication	9 (10.0)
​​​​​​​^2^Other	6 (6.7)
CKD	n=53
Hypertension	19 (35.8)
Diabetes mellitus	18 (34.0)
HIV associated nephropathy	8 (15.1)
Non-steroidal anti-inflammatory drugs	6 (11.3)
Idiopathic	5 (9.4)
​​​​​​​^3^Other	4 (7.5)
AoCKD	n=49
Sepsis	18 (36.7)
Urosepsis	9 (18.4)
Pneumonia	4 (8.2)
Skin and soft tissue infections	3 (6.1)
Bacteremia	2 (4.1)
Congestive cardiac failure	15 (30.6)
​​​​​​​^4^Obstructive nephropathy	8 (16.3)
Gastroenteritis	5 (10.2)
Herbal medication	3 (6.1)

**Figure 1:**
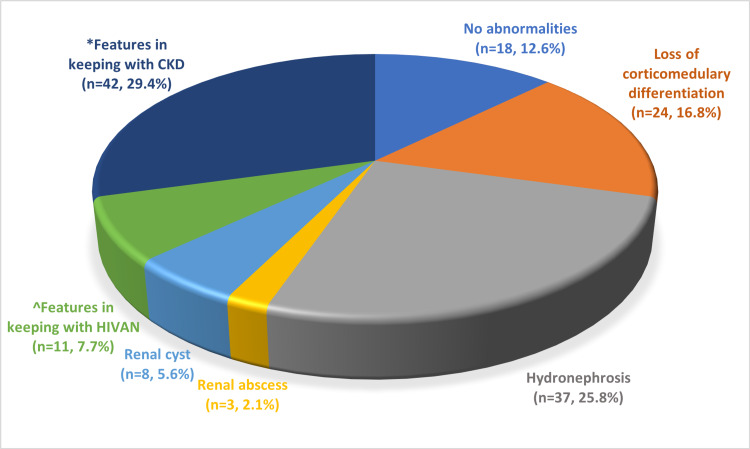
Findings of the 143 subjects in whom renal ultrasonography was performed *Small and hyperechoic kidneys ^Large and hyperechoic kidneys in subjects with HIV

Renal ultrasonography was performed on 143 (74.5%) subjects, with 125 (87.4%) displaying abnormal sonographic features. Figure 1 illustrates the various ultrasonographic findings.

## Discussion

Renal dysfunction, especially AKI in patients admitted to the intensive care unit and the adult acute renal unit, has been widely studied in multiple small and large-scale studies [12,15]. However, its incidence in the ED is poorly defined, largely due to differences in case definition and case-mix [16]. To the best of our knowledge, this is the first study describing the presentation of patients with renal dysfunction presenting to a tertiary academic hospital ED in Johannesburg.

The demographic features of patients with renal dysfunction in this study were in accordance with previous studies in Africa. A male predominance was noted in this study (56.3%). Comparatively, an ED-based study conducted by Sylvanus et al., in Dar es Salaam, Tanzania reported that males comprised 75.3% of patients with renal failure [17]. Dlamini et al., in Cape Town, South Africa, and Kaze et al., in Yaunde, Cameroon, reported similar rates of 58.5% and 60% in males, respectively [16,18]. The median age of subjects in this study (49.5 years; IQR 38.8-63.0 years) was similar to the findings of Sylvanus et al. (49 years; IQR 32-66 years) [17].

More than a third (37.5%) of study subjects were HIV-positive, which is much higher than the reported national prevalence of HIV (approximately 20%) [19]. Comparatively, Vachiat et al. and Dlamini et al. reported the prevalence of HIV as 14.8% and 20.5%, respectively [12,16]. HIV-infected individuals are more prone to renal dysfunction due to various factors, including higher rates of sepsis (primarily related to opportunistic infections), diarrhea, and direct infection of the kidney with HIV itself (HIV-associated nephropathy). Other mechanisms or causes of renal dysfunction in HIV-infected individuals include immune complex-mediated kidney disease, non-collapsing focal segmental glomerulosclerosis, and prolonged exposure to certain classes of antiretroviral therapy [20,21].

The proportion of patients with AKI (46.9% vs 56%), CKD (27.6% vs 23%), and AoCKD (25.5% vs 21%) was very similar to the findings of Vachiat et al. [12]. The incidence of AKI of 1.2% (90/7442) in this study was low compared to other studies. The study by Dlamini et al. reported an incidence of 3.4% among 10 750 hospital admissions [16], whereas a study conducted in Alabama, USA reported that the incidence of AKI was 22.7% among 19,249 hospitalized patients [22]. The incidence of AKI is much higher in ICU patients. A study conducted at an academic hospital ICU in northeast Brazil reported the incidence of AKI as 32.9% [6]. Factors such as the use of nephrotoxic drugs, progression of presenting illness, nosocomial sepsis, hypotension, mechanical ventilation, and intravenous contrast media administration all predispose hospitalized patients to developing AKI as compared to ED patients [23]. This is a likely reason for the much higher rates of AKI reported in these studies.

In this study, the incidence of CKD was 0.7% (53/7442), with comorbid hypertension and diabetes mellitus being reported by 35.8% and 34.0% of subjects, respectively. A study conducted in São Paulo, Brazil reported that the incidence of CKD was 12.7%, with comorbid hypertension and diabetes being reported by 75.2% and 49.5% of patients, respectively [24]. Another study conducted in Gaborone, Botswana, reported that the incidence of CKD was 13.5%, with hypertension and diabetes being present in 67.4% and 25.6% of patients, respectively [25]. Unlike our study that was conducted in the ED, these studies enrolled hospitalized patients, which is a likely explanation for the higher reported incidence of CKD in these studies.

Sepsis was the most common precipitant of both AKI (42.2%) and AoCKD (36.7%) in this study. The rates of sepsis were lower compared to the two other South African-based studies by Vachiat et al. and Dlamini et al., who reported that sepsis was the precipitant for AKI in 59.4% and 60.7% of cases, respectively [12,16]. In other studies, sepsis as a precipitant of AKI ranged between 20.8% and 52% of cases [13,15,26,27]. Compared to our study, where urosepsis was the most common source of sepsis in both subjects with AKI (20.0%) as well as those with AoCKD (18.4%), a study by Brown et al. in the United States of America reported that pneumonia (11.5%) was the most common source of sepsis in patients with AKI [26]. Compared to other studies where obstructive uropathy accounted for between 7.6% and 46.5% of cases of AKI [28,29], in our study obstructive uropathy accounted for 27.8% of cases of AKI and 16.3% of cases of AoCKD.

In our study, 39.6% of subjects required renal dialysis, which was similar to the findings of Sylvanus et al. in Tanzania (42%) [17]. With regards to mortality, Vachiat et al. and Dlamini et al. reported rates of 42.0% and 40.1%, respectively [12,16], which were higher than the 30.7% mortality rate in our study.

The relatively high proportion of subjects with renal dysfunction and concurrent HIV, sepsis, urinary tract obstruction, hypertension, diabetes mellitus, and congestive cardiac failure were significant findings of our study. Factors such as cultural practices, local healthcare protocols, clinical settings (private versus public healthcare), and local HIV prevalence may limit the generalizability of our data. Despite the above limitations, our study is still valuable because it highlights factors associated with renal dysfunction in patients presenting to the ED.

## Conclusions

In this ED-based study, AKI accounted for approximately half the number of cases presenting with renal dysfunction, while CKD and AoCKD accounted for approximately a quarter of cases each. Overall, mortality among patients with renal dysfunction was high, with close to a third demising prior to hospital discharge. HIV, sepsis, urinary tract obstruction, hypertension, diabetes mellitus, and congestive cardiac failure were frequent comorbidities. Therefore, clinicians should adopt a low threshold to screen for renal dysfunction in patients presenting to the ED with these comorbidities.

## References

[1] Joslin J, Ostermann M (2012). Care of the critically ill emergency department patient with acute kidney injury. Emerg Med Int.

[2] Acosta-Ochoa I, Bustamante-Munguira J, Mendiluce-Herrero A, Bustamante-Bustamante J, Coca-Rojo A (2019). Impact on outcomes across KDIGO-2012 AKI criteria according to baseline renal function. J Clin Med.

[3] Hoste EA, Kellum JA, Selby NM (2018). Global epidemiology and outcomes of acute kidney injury. Nat Rev Nephrol.

[4] Hill NR, Fatoba ST, Oke JL, Hirst JA, O'Callaghan CA, Lasserson DS, Hobbs FD (2016). Global prevalence of chronic kidney disease - a systematic review and meta-analysis. PLoS One.

[5] (2012). KDIGO Clinical Practice Guideline for acute kidney injury: notice. Kidney Int Suppl (2011).

[6] Santos PR, Monteiro DL (2015). Acute kidney injury in an intensive care unit of a general hospital with emergency room specializing in trauma: an observational prospective study. BMC Nephrol.

[7] Li PK, Burdmann EA, Mehta RL (2013). Acute kidney injury: global health alert. Kidney Int.

[8] (2012). KDIGO Clinical Practice Guideline for acute kidney injury: references. Kidney Int Suppl (2011).

[9] Kazancioğlu R (2013). Risk factors for chronic kidney disease: an update. Kidney Int Suppl (2011).

[10] Hsu RK, Hsu CY (2016). The role of acute kidney injury in chronic kidney disease. Semin Nephrol.

[11] Hsu CY, Ordoñez JD, Chertow GM, Fan D, McCulloch CE, Go AS (2008). The risk of acute renal failure in patients with chronic kidney disease. Kidney Int.

[12] Vachiat AI, Musenge E, Wadee S, Naicker S (2013). Renal failure in HIV-positive patients: a South African experience. Clin Kidney J.

[13] Arendse CG, Wearne N, Okpechi IG, Swanepoel CR (2010). The acute, the chronic and the news of HIV-related renal disease in Africa. Kidney Int.

[14] von Elm E, Altman DG, Egger M, Pocock SJ, Gøtzsche PC, Vandenbroucke JP (2007). Strengthening the reporting of observational studies in epidemiology (STROBE) statement: guidelines for reporting observational studies. BMJ.

[15] Wijewickrama ES, Ratnayake GM, Wikramaratne C, Sheriff R, Rajapakse S (2014). Incidences and clinical outcomes of acute kidney injury in ICU: a prospective observational study in Sri Lanka. BMC Res Notes.

[16] Dlamini TA, Heering PJ, Chivese T, Rayner B (2017). A prospective study of the demographics, management and outcome of patients with acute kidney injury in Cape Town, South Africa. PLoS One.

[17] Sylvanus E, Sawe HR, Muhanuzi B, Mulesi E, Mfinanga JA, Weber EJ, Kilindimo S (2019). Profile and outcome of patients with emergency complications of renal failure presenting to an urban emergency department of a tertiary hospital in Tanzania. BMC Emerg Med.

[18] Kaze FF, Ekokobe FE, Halle MP, Fouda H, Menanga AP, Ashuntantang G (2015). The clinical pattern of renal diseases in the nephrology in-patient unit of the Yaounde General Hospital in Cameroon: a five-year audit. Pan Afr Med J.

[19] (2022). HIV and AIDS in South Africa. https://www.avert.org/professionals/hiv-around-world/sub-saharan-africa/south-africa.

[20] Naicker S, Rahmanian S, Kopp JB (2015). HIV and chronic kidney disease. Clin Nephrol.

[21] Swanepoel CR, Atta MG, D'Agati VD (2018). Kidney disease in the setting of HIV infection: conclusions from a Kidney Disease: Improving Global Outcomes (KDIGO) Controversies Conference. Kidney Int.

[22] Wang HE, Muntner P, Chertow GM, Warnock DG (2012). Acute kidney injury and mortality in hospitalized patients. Am J Nephrol.

[23] Singh TB, Rathore SS, Choudhury TA, Shukla VK, Singh DK, Prakash J (2013). Hospital-acquired acute kidney injury in medical, surgical, and intensive care unit: a comparative study. Indian J Nephrol.

[24] Pinho NA, Silva GV, Pierin AM (2015). Prevalence and factors associated with chronic kidney disease among hospitalized patients in a university hospital in the city of São Paulo, SP, Brazil. J Bras Nefrol.

[25] Rwegerera GM, Bayani M, Taolo EK, Habte D (2017). The prevalence of chronic kidney disease and associated factors among patients admitted at princess marina hospital, Gaborone, Botswana. Niger J Clin Pract.

[26] Brown JR, Rezaee ME, Marshall EJ, Matheny ME (2016). Hospital mortality in the United States following acute kidney injury. Biomed Res Int.

[27] Bouchard J, Acharya A, Cerda J (2015). A prospective international multicenter study of AKI in the intensive care unit. Clin J Am Soc Nephrol.

[28] Caddeo G, Williams ST, McIntyre CW, Selby NM (2013). Acute kidney injury in urology patients: incidence, causes and outcomes. Nephrourol Mon.

[29] Hamdi A, Hajage D, Van Glabeke E (2012). Severe post-renal acute kidney injury, post-obstructive diuresis and renal recovery. BJU Int.

